# Both “Vitamin L for Life” and “One Milligram of Satan”: A Multi-Perspective Qualitative Exploration of Adjuvant Endocrine Therapy Use after Breast Cancer

**DOI:** 10.3390/curroncol28040227

**Published:** 2021-07-05

**Authors:** Kirsti I. Toivonen, Devesh Oberoi, Kathryn King-Shier, Katherine-Ann L. Piedalue, Joshua A. Rash, Linda E. Carlson, Tavis S. Campbell

**Affiliations:** 1 Department of Psychology, University of Calgary, Calgary, AB T2N 1N4, Canada; kirsti.toivonen@ucalgary.ca; 2 Division of Psychosocial Oncology, Department of Oncology, Cumming School of Medicine, University of Calgary, Calgary, AB T2N 1N4, Canada; devesh.oberoi@ucalgary.ca (D.O.); klpiedal@ucalgary.ca (K.-A.L.P.); 3 Faculty of Nursing, University of Calgary, Calgary, AB T2N 1N4, Canada; kking@ucalgary.ca; 4 Department of Psychology, Memorial University of Newfoundland, St. John’s, NL A1C 5S7, Canada; jarash@mun.ca

**Keywords:** qualitative, breast cancer, persistence, adjuvant endocrine therapy

## Abstract

Adjuvant endocrine therapy (AET) is recommended after hormone receptor-positive breast cancer to reduce risk of recurrence, but adherence is sub-optimal in many women. Behavioral interventions have been ineffective in improving adherence rates to AET. This qualitative descriptive study investigates factors that support women in AET use and suggestions for interventions to improve AET use and management. Interviews with women who persisted with AET (*n* = 23), women who discontinued AET (*n* = 15), and healthcare providers (HCPs; oncologists, oncology residents, and pharmacists; *n* = 9) were conducted, transcribed, and described using thematic analysis. Data collection stopped once saturation occurred (i.e., no new codes or themes emerged during interviews). Two researchers created codes and developed themes in an iterative process; a third researcher verified the representativeness of final themes. This study was approved by the Health Research Ethics Board of Alberta (ID: HREBA.CC-17-0513). Women who persisted described being prepared for side effects and having self-management strategies, strong rationale for AET use, supportive HCPs, and available resources as relevant factors. Women who discontinued described feeling overwhelmed by side effects, information needs, drawbacks of AET, helpful/unhelpful experiences with HCPs, and contextual factors as relevant to their discontinuation. HCPs described health system-related and patient-related barriers, side effect management, and patient-provider interactions as relevant to supporting AET use. The considerable overlap in themes among the three groups suggests broad recognition of salient factors relevant to AET use and that associated strategies to improve use may be acceptable to patients and providers alike. Factors supporting AET use could include the following: education (which may be necessary but insufficient), developing a strong personal rationale for use, being prepared for side effects, having side effect management strategies, reciprocal communication between patients and HCPs, and accessible resources.

## 1. Introduction

Adjuvant endocrine therapy (AET), including tamoxifen and aromatase inhibitors (AIs), is recommended for up to 10 years following primary treatment for hormone receptor-positive breast cancer to reduce the risk of recurrence [1]. Despite the efficacy of AET, it is estimated that 79% of women adhere within the first year of use, decreasing to 56% by the fifth year [2]. Several factors have been identified as correlates of adherence, including lower side effect severity, greater self-efficacy, belief in the necessity of AET, and positive relationships with healthcare providers (HCPs) [3,4]. Sub-optimal AET use is an important clinical issue as non-adherence and discontinuation are associated with a 49% and 26% increase in mortality, respectively [5]. Further, interventions to date have been largely unsuccessful in improving adherence to AET among breast cancer survivors [6,7].

Interventions that can support women’s adherence to AET have the potential to directly improve the lifespan of breast cancer survivors. Best practices for behavioural intervention development recommend starting with a thorough understanding of the clinical problem of interest in order to map out plausible pathways through which a behavioural treatment can solve the problem and aid in the selection of meaningful intervention targets [8]. Qualitative methods are well-suited to inform the development of behavioural interventions [9] by deepening understanding of phenomena being studied, helping identify participant-centered strategies, and highlighting the acceptability of potential intervention components [8].

Qualitative studies can offer information about the nuanced ways in which women’s experiences with AET affect adherence, beyond results from quantitative studies. For example, reviews of quantitative studies demonstrate an association between more severe side effects and lower adherence, while qualitative studies can provide plausible explanations for the association. Women in such studies have described how AET side effects impair their quality of life and relationships with others [10,11], make them feel older or unfeminine [12], and prevent them from feeling normal [11,13]. Studies have also highlighted how side effects are even more difficult to manage when unexpected [11,13,14,15]. 

The purpose of this study is to understand factors associated with AET use and develop a deeper understanding about specific intervention components participants would find helpful in supporting AET use. This study contributes to the literature in two important ways. First, women who persisted with AET, women who discontinued AET, and HCPs are included, providing the opportunity to compare viewpoints. Multiple perspectives may provide a more holistic understanding of barriers, facilitators, and potential strategies to pursue. Second, this study is designed to inform intervention development by explicitly asking women who persisted and HCPs what strategies helped them or their patients persist with AET, and asking women who discontinued what support or resources they think would be/have been helpful. Insight about fruitful targets for intervention development suggested by patients and healthcare providers may lead to the development of interventions that are meaningful, feasible, and acceptable.

## 2. Methods

This qualitative descriptive study was approved by the Health Research Ethics Board of Alberta (ID: HREBA.CC-17-0513). All participants provided written informed consent. Anonymity was ensured by removing identifying information from transcripts and omitting potentially revealing information in quotes. Consolidated criteria for reporting qualitative research were followed [16].

### 2.1. Participants

Three groups were included. Groups one and two included English-speaking women who were diagnosed with breast cancer and prescribed AET. Women in group one reported plans to persist with AET (even if they had taken breaks or switched medications) despite at least some self-reported difficulty with AET. Women in group two reported discontinuing or not initiating AET. Two women who discontinued AET solely for pregnancy were excluded; there were no other exclusion criteria. The third group was English-speaking healthcare providers (HCPs) including pharmacists, oncologists, and oncology residents. There were no exclusion criteria based on duration of practice or any other parameters.

### 2.2. Recruitment Process 

Women who persisted with AET were recruited from the participant pool of a study examining predictors of AET adherence in Alberta [17]. Women who persisted with AET despite at least some difficulty were purposively sampled [18], such that women who described having no issues with AET during recruitment for the prior study were not invited to participate. Women who discontinued AET were recruited from the Alberta Cancer Registry via an invitation letter. This letter was mailed to a random sample of 1000 women diagnosed with hormone-receptor positive breast cancer within the past 1–8 years and asked for women who specifically discontinued AET to contact researchers. A female Doctoral candidate in clinical psychology (K.I.T.) screened potential participants who responded to the invitation about reason for stopping. One woman who discontinued because of a specific allergy to AET was excluded as this was unlikely to be broadly relevant. Finally, HCPs were recruited via a presentation delivered after oncology rounds at an Alberta hospital and through an email request circulated to the oncology department. Two HCPs in British Columbia heard about the study through word-of-mouth and were also included. All participants were informed that the purpose of the study was to understand factors associated with AET use to inform intervention development during the recruitment process. Recruitment occurred between November 2018 and March 2020 and stopped once saturation occurred (i.e., no new codes or themes arose during interviews [19]). No participants withdrew after enrolment and participation was not incentivized.

### 2.3. Data Collection

K.I.T. recruited all participants and conducted all interviews. K.I.T. had prior contact with women who persisted with AET through conducting the study from which they were recruited, but had no pre-existing relationships with participants from other groups. Interviews occurred at the researcher’s office, participant’s home, or via telephone based on participant preference. Demographic information was collected before the interviews. All interviews were conducted with individuals rather than groups to enable understanding of participants’ personal experiences and allow privacy to discuss potentially sensitive issues. Interviews were semi-structured, following a template of open-ended questions (Appendix A) with additional probes added as interviews progressed. Interviews were audio recorded then transcribed verbatim by research assistants or a professional transcription service who signed confidentiality agreements. K.I.T. verified all transcripts against the original audio recordings for accuracy. Interviews ranged from 12 to 76 minutes with breast cancer survivors (M = 39.26, SD = 13.84) and 12 to 37 minutes with HCPs (M = 19.11, SD = 8.43).

### 2.4. Data Analysis

Thematic analysis was used to organize and categorize patterns within the data, using an inductive, descriptive approach [20,21]. Only sections of transcripts explicitly relevant to adherence were coded given that this project was meant to inform interventions to improve adherence. Two researchers (K.I.T. and D.O.) read the transcripts, created codes, and noted patterns in the data [20]. They discussed potential themes and then re-read transcripts to refine themes multiple times in an iterative process. A third researcher (K.-A.L.P.) also read the transcripts and verified the representativeness of the final themes. Themes from each group were identified separately to allow examination of the extent to which similar themes emerged across groups. NVivo software (QSR International Pty Ltd., Melbourne, Australia; Version 12, 2018) was used to sort and compile codes. 

Three methods were used to enhance the trustworthiness of the data. First, an audit trail was maintained that tracked decisions around the assignment of quotes to codes and assignment of codes to themes through each iteration of the analysis [22]. Second, the research team compared the themes to descriptions and quotes to verify the accuracy of results [23]. Finally, member checking was used [22], such that all participants were provided the opportunity to review the results. Fifteen participants responded, all of whom verified that the results accurately represented their experience.

## 3. Results

The average age of women who persisted (*n* = 23) was 56 and women who discontinued (*n* = 15) was 64 years. The groups were on average 2.6 years and 3.7 years post-diagnosis, respectively. Most women were White and had stage I or II cancer. See Table 1 for demographic information. Nine HCPs participated, including three pharmacists, two medical oncologists, and four oncology residents. While duration of practice is not reported to protect confidentiality, pharmacists and oncologists had worked in their fields for at least five years. Within each group of participants, overarching themes and relevant sub-themes were identified; descriptions and representative quotes are provided subsequently.

### 3.1. Women Who Persisted 

Among women who persisted, factors that supported AET use were classified under three broad themes: side effects, rationale for use, and experiences with the healthcare system. Additional quotes are outlined in Table 2.

#### 3.1.1. Side Effects 

*Preparedness for side effects*. The experience of side-effects was near-universal among women who persisted and impacted their quality of life by interfering with hobbies, valued activities, and relationships with others. Although side effects were difficult to manage, nearly half of women who persisted expected side effects based on information relayed by HCPs or other breast cancer survivors. Expectation of side effects reduced their impact somewhat by helping participants mentally prepare:

*“I knew there was going to be side effects, because everybody kept saying that. And I think it was a frame of mind I put myself in that I was going to power through it.”*

(Participant, age 51)

In addition, participants who had pre-existing medical conditions indicated that they were used to experiencing and managing symptoms similar to AET side effects, such as pain. Five participants indicated that they were not bothered by side effects such as hot flashes as they expected to experience them with age. One woman, anticipating joint pain, increased her level of exercise before starting AET to prepare her body. In contrast, the few participants who did not experience side effects described using AET as a relatively simple decision. 

*Management strategies*. Women who persisted were well-equipped with strategies to manage side effects. They identified behavioural strategies and pharmacological options; however, half of participants disliked the idea of treating medication side effects with more medications. Of behavioural strategies, exercise was consistently endorsed as helpful for managing joint and muscle pain. Additionally, participants identified that exercise had multiple benefits that extended from specific side effects to general well-being (e.g., mental health, sleep). Two participants felt strongly that exercise should be considered part of cancer care, with one suggesting that exercise equipment be incorporated in oncology clinic waiting rooms to facilitate access and make use of long wait times.

*“I think it could come down to almost, like, you need to be prescribed exercise.”*

(Participant, age 60)

Five participants described troubleshooting the management of side effects by taking medications at different times, with or without meals, or trying natural health products to find effective strategies. For example, one woman discovered that wheatgrass helped her joint pain through the collection of data about her strategies and symptoms. Several participants noted that they were able to manage side effects such as fatigue through modifying their lifestyle (e.g., pacing, taking breaks, or reducing intensity of activities). One-third of the women who persisted identified the importance of attitudes such as acceptance or positivity for coping with side effects. However, another cautioned that focusing on the importance of attitude could be construed as patient-blaming, as if suggesting unfavorable outcomes are deserved in the absence of the “right” attitude. Women provided several suggestions for supporting AET side effect management, such as having a dedicated HCP available for guidance around side effects or dedicated resources such as group education sessions. Five women noted that they would like support from other women who had been prescribed AETs and three specifically wanted to hear tips from other women about AET side effect management.

*“Being able to talk to other people about what worked and what didn’t, the whole question of, what can I expect? For instance, with bone pain, a lot of people talk about how Claritin, which is an anti-allergy pill, apparently is magical for bone pain, and so I haven’t used it, but it’s good to know.”*

(Participant, age 51)

#### 3.1.2. Rationale for Use

Nearly all women who persisted understood that AET affected estrogen to prevent cancer recurrence and noted that this knowledge gave them peace of mind to persist with AET. Women who persisted identified several reasons that supported their decision to use, while also recognizing the limitations of AET. Half the participants feared cancer recurrence and wanted to do everything they could to live, though a third acknowledged that AET was not guaranteed to prevent recurrence. Three women explicitly identified taking AET in an attempt to avoid living with regret had they not taken AET and experienced a recurrence:

*“The bigger piece for me was, what would it cost me if I abandon it and [the cancer] was to come back? Would I at least feel comfort that I did everything, or would I be plagued or haunted by, I abandoned early because I was uncomfortable?”*

(Participant, age 41)

Other reasons supporting AET use included trusting an oncologist’s recommendation, trusting research, and wanting to live for family members (especially children). Despite having knowledge about AET, women who persisted wanted more detailed information about AET and, when they did not receive this from HCPs, they often sought it themselves from the Internet. Participants recognized that there were risks to AET, such as a risk of developing uterine cancer or osteoporosis, but they were willing to tolerate risks for AET’s potential life-saving benefits. One participant referred to her anastrozole as both “*one milligram of Satan*” and “*vitamin L… for life*”, which captured a common sentiment that participants could simultaneously derive comfort from AET while suffering from its side effects.

#### 3.1.3. Experiences with the Healthcare System 

*Interactions with healthcare providers*. Participants noted that their interactions with HCPs, particularly oncologists, mattered and were important enough to influence their decision to persist with AET. Women especially valued oncologist’s knowledge, as well as oncologists listening to them, communicating with empathy, and treating them as individuals.

*“I wouldn’t have stayed on it probably if it wasn’t for [my oncologist]. I just really respect his knowledge and he was just so caring and thoughtful and sensitive; and had a little bit of a humor spin to things. He was amazing and I could really tell him anything.”*

(Participant, age 60)

One participant recounted how she lost a prescription refill and would have missed three months rather than ask for another refill had her pharmacist not been so kind and empathetic. Women also appreciated HCPs being responsive to their concerns, for example, by trying to give them strategies for managing their side effects or connecting them to other resources.

Conversely, women who persisted expressed frustration at instances where they felt that HCPs did not listen to them, were interpersonally cold, were dismissive of their concerns, or spent little time with them during appointments. One participant reported that her frustration with feeling ignored and very brief appointment times caused her to stop consulting with her oncologist about AET. However, participants recognized that institutional pressures existed (e.g., high caseloads) that limited their appointment times. One participant struggled with vaginal atrophy and was concerned about the role AET might play in the progression of her symptoms, but perceived HCPs as being uncomfortable discussing such changes and their impact on her sex life.

*Availability of resources*. Half of the participants identified that resources were locally available to support breast cancer survivors using AET. These included support groups, educational sessions, tailored programming, and exercise classes specifically geared towards cancer survivors. Some specifically identified a local non-profit community-based clinic for breast cancer patients as being extremely helpful; this clinic offers medical care, interdisciplinary supportive resources, and extended meetings with physicians. The fact that AET could be mailed to their home was appreciated and one woman noted that this allowed her to persist:

*“If I had to go to the hospital to get [AET], I’m not sure I would because that parking is ridiculous.”*

(Participant, age 66)

Although nearly all women were aware that additional resources were available, not everyone availed of them, for example, if they did not need extra support or felt the resources would not be helpful or resonate with them. For example, eight women specifically disliked support groups and did not want to share or be in a group environment.

*“I don’t go to groups, because–I’m not depressed. I have a feeling [laughing] if I went to those groups, maybe I would get depressed.”*

(Participant, age 70)

### 3.2. Women Who Discontinued

Contributing factors to discontinuing use of AET were classified under four themes: overwhelming side effects, reluctance, experiences with healthcare providers, and contextual factors. Additional quotes are presented in Table 3.

#### 3.2.1. Overwhelming Side Effects

Almost all women who discontinued AET did so because of side effects (e.g., severe arthralgia, itching, fatigue, nausea, headaches, and hot flashes), which negatively impacted quality of life and relationships with others, as well as ability to work, sleep, use their hands, or engage in valued activities. The impact was so great that participants ultimately reached a breaking point where they decided to prioritize quality of life over longevity. Being unprepared for side effects compounded their effect. While it was common for participants to say that side effects were worse than they expected; some wondered if information about side effects was purposely downplayed. At worst, four participants misinterpreted side effects as signs of cancer recurrence or metastasis, which frightened them.

*“I’m going between oncology and my family doctor saying, “What’s wrong with me, I don’t feel good”–and then starting the fear of, “It must be in my bones, my bones hurt, the cancer must be back”. So I kind of went into a tailspin.”*

(Participant, age 58)

Further, women who discontinued AET described being unable to manage their side effects despite efforts to do so. While half of participants tried pharmacological strategies for side effects, they found medications ineffective, had even more secondary side effects (e.g., gastro-intestinal issues from analgesics), or worried about long-term effects of medication use. They also tried non-pharmaceutical side effect management strategies such as dietary changes, vitamins, and exercise, which were also ineffective. Others noted that they would have liked to try strategies such as exercise, but faced barriers such as a lack of time or access to equipment, living far from classes, or the timing of classes conflicting with work schedules. Over half of the women who discontinued described trying to persist with AET through medication switches or breaks, rejecting the notion that they “gave up” easily. However, breaks from AET would confirm to them how poorly it made them feel. Overall, women who discontinued wished that they had had effective options for side effect management.

#### 3.2.2. Reluctance

*Information needs*. About two-thirds of women who discontinued understood that AET reduced cancer risk by affecting estrogen or hormones in general; however, one-third stated that they did not know how AET worked beyond helping prevent cancer. Some women felt that, because they did not understand AET, their decision to initiate it was not truly informed.

*“I wasn’t quite sure how the hormone blocker worked. I didn’t quite understand that I would be having decreased estrogen and then all the bad stuff that goes with that.”*

(Participant, age 58)

Women noted that they wanted more information about AET from their HCPs, including rationale for use, mechanisms by which it worked, and specific information about their personal risk of recurrence. However, when women had unmet information needs, they often turned to the Internet as a source of information, which included statistics websites, online forums for breast cancer survivors, or general online searches. Women provided several suggestions for supporting information needs about AET. These included providing information in a centralized source (e.g., website affiliated with the cancer centre), ensuring information is written (as a memory aid) and explained in lay terms, or providing avenues for women to hear from other breast cancer survivors about experiences with AET or tips for managing side effects.

*Drawbacks of AET*. Women who discontinued identified several drawbacks to AET, such as the long-term commitment, lack of guaranteed effectiveness, and fear of long-term adverse effects such as bone loss. Two participants were unwilling to initiate AIs because they feared the risk of developing osteoporosis, especially because they saw the impact of osteoporosis on loved ones.

*“I guess it was a matter of which are you more afraid of, recurrence of the cancer or osteoporosis? And I guess I picked osteoporosis.”*

(Participant, age 73)

Five women who discontinued explicitly stated they believed AET was of low personal benefit (e.g., because they had an earlier-stage cancer), and one described tamoxifen as “*a pill for every ill*”, considering it too generic for her specific disease characteristics. Four participants simply disliked pharmaceuticals or were generally wary of pharmaceutical companies. Two noted that they preferred a natural or holistic approach to cancer care (including focus on diet and supplements to support immune function) and felt comfortable with having discontinued AET because they were currently using complementary therapies.

#### 3.2.3. Experiences with Healthcare Providers

Women who discontinued noted how experiences with HCPs and the healthcare system were relevant to their medication use. Several women who discontinued noted that their HCPs worked hard to support them in AET use by offering management strategies, suggesting breaks, and managing medication switches. Women who discontinued typically gave great effort to persist with AET, including trying multiple medications or trialing them multiple times and, in some cases, it was ultimately HCPs who suggested stopping to alleviate the participant’s suffering. Three participants expressed gratitude towards HCPs for having made this decision for them.

*“And by her saying, “That’s it, enough, no more”, I kind of felt relieved. I’m scared, but I’m relieved and I just came to terms with it.”*

(Participant, age 58)

Participants appreciated when HCPs listened to them and were empathetic and compassionate. It was important to them that they had autonomy over their medical decisions and appreciated when HCPs allowed them to have an active role in decision-making around AET. 

Conversely, participants were frustrated when they perceived that HCPs were unwelcoming of questions, or when they felt rushed during appointments. Women also disliked when they perceived HCPs as unresponsive. Examples of this included if participants’ calls went unreturned, if they indicated bothersome symptoms on routine surveys that were not followed up on, or if they disclosed concerns about side effects directly to HCPs but received no suggestions or referrals. Some women expressed feeling too guilty to raise concerns about AET side effects to their oncologists, as they perceived their needs to be less important than those undergoing primary cancer treatment.

#### 3.2.4. Contextual Factors

Some of the participants who had pre-existing medical conditions or pain, lived alone, were older, or had to work out of financial necessity had more difficulty justifying AET use in light of their significant side effects. Two described how they had tried to hide the fact that they were struggling with AET, but eventually it became apparent and affected their work or relationships. Having to work out of financial necessity was difficult for participants who had limited energy and stamina due to AET side effects. It also prevented them from being able to access support and resources that they felt might have been helpful, for example, when resources were only available during daytime working hours. Women who described themselves as being at low-risk for cancer recurrence based on lower cancer stage did not consider AET to be worth enduring the side effects for potentially little reduction in the risk of recurrence.

*“It would also be different if the stage of my cancer was different. But being a stage one, I felt that the odds were in my favour.”*

(Participant, age 67)

Alternatively, women identified personal attitudes of acceptance, conviction, and sense of humour as factors that helped them persist with AET for the time that they did. One woman who had cancer previously stated that she worked even harder to persist with AET the second time around, though ultimately decided to discontinue because of side effects.

### 3.3. Healthcare Providers 

Factors related to AET use described by HCPs encompassed three themes: barriers at patient- and system-levels, side effect management, and patient-provider interactions. Table 4 includes additional quotes.

#### 3.3.1. Barriers at Patient- and System-Levels

HCPs identified barriers to AET use that patients face, as well as system-level barriers that HCPs face in supporting patient adherence to AET. At the patient-level, HCPs noticed that patients could have pre-existing biases against AET owing to a general dislike of medications, preference for a natural option, not wanting to be reminded of the cancer, reading misinformation, or hearing about others’ negative experiences with AET.

*“There is a lot of misinformation out there. And that can be either through the internet or through a relationship with somebody that they know who had a bad outcome with one of the drugs. So they’ll get a very skewed view on what the medication’s all about, or the potential harms.”*

(Oncologist)

Conversely, one HCP acknowledged that the Internet can be a helpful source of information for patients. Several HCPs noted that patients could face practical barriers such as driving to appointments for prescription refills and pharmacies, particularly if they live in rural areas. In Alberta, AET is free of cost from cancer care pharmacies and can be delivered by mail for free. However, HCPs identified that, in other places where mailed prescriptions are not offered, practical issues such as driving and parking when refilling prescriptions could make cost a barrier.

System-level barriers to supporting patient adherence included limited time with individuals, which may reduce an HCP’s ability to thoroughly understand and address patient concerns. Similarly, large gaps between appointments due to high clinical loads relative to number of staff could make continuity of care difficult. One pharmacist noted that they would have liked to have a call-back program implemented to target poor adherence, but that there simply were not enough staff. Another pharmacist identified discharge to family physicians as a common point along the care trajectory where adherence is at risk of dropping; they suggested sharing information or resources about AET with family physicians through primary care networks to help combat this issue. HCPs additionally noted the inherent difficulty in identifying and supporting people who do not adhere or persist because they do not return to clinics or call for refills.

#### 3.3.2. Side Effect Management 

Similar to breast cancer survivors, HCPs identified side effects as one of the greatest barriers to AET use, with one stating that they had not seen anyone discontinue for reasons other than side effects. This underscored the importance of their role in helping women to manage their side effects. HCPs identified the importance of having behavioural interventions and pharmacological options to manage potential side effects; two explicitly noting that they preferred the former as the first-line approach. In particular, HCPs endorsed exercise for side effects such as joint pain, but also emphasized the importance of exercise as a part of healthy living more broadly.

*“I would hope that if there were going to be an effort made to improve adherence to medication taken over the long-term, that it could be envisioned as something broader than that. That it did also include advice on healthy living which I think may provide numerically as much benefit as the hormone treatments and maybe more.”*

(Oncologist)

HCPs identified pharmacological options for more severe side effects, such as antidepressants for bothersome hot flashes. Some HCPs encountered barriers to pharmacological management, such as patients disliking stigma attached to an antidepressant being used for hot flashes, or not wanting to treat medication side effects with more medication. Conversely, one HCP noticed that some patients favour a pharmacological solution for side effects, as it can be relatively easy to use and fast-acting. Other options for managing side effects included switching medication, especially between AIs. HCPs also noted that community resources that could help breast cancer survivors with AET management existed, but were often underutilized. Streamlining information about resources, providing tailored resources, and normalizing their use by offering them to every person were strategies suggested by HCPs to improve uptake.

#### 3.3.3. Patient–Provider Interactions 

HCPs identified communication as an important factor contributing to AET use, with the belief that communication must be reciprocal to be effective. For their part, HCPs emphasized providing a balanced perspective about the risks and benefits of AET use and encouraged patients’ autonomy in decision-making based on all available information. All HCPs identified education as a primary tool in supporting AET use, which included proactive discussion of side effects. HCPs acknowledged that each patient has multiple factors affecting their medication use, different responses to medications, and different information needs–which necessitates an individualized approach to care.

*“It’s not a one size fits all. It’s a communication and consultation process with each individual patient, depending on their disease characteristics, goals, beliefs, comorbidities, age. It’s not something you can predict before you’re meeting with that individual.”*

(Oncologist)

HCPs noted a reduced ability to support patients in AET use when patients withheld information from them. For example, three HCPs suspected that patients may underreport symptoms, perhaps owing to guilt about extending appointment times or embarrassment about symptoms. HCPs also wondered if women hesitate to report symptoms (such as hair loss or thinning) that they worry may be perceived as trivial. To mitigate this, HCPs encouraged an open line of communication and asked open-ended questions in a non-judgmental manner. Additional strategies HCPs used to support AET use included enlisting support from interdisciplinary HCPs, conducting follow-up calls, close initial follow-ups, simplifying messaging or focusing on one issue at a time, troubleshooting with patients as needed, and trying to empower patients by providing options for self-management.

### 3.4. Comparison Across Groups

There was considerable overlap in themes identified among the three groups (see Table 5). The most salient factor recognized by all groups was the adverse impact of side effects and the importance of effective side effect management strategies. However, women who persisted tended to describe being prepared and having effective strategies, while women who discontinued felt less prepared did not have effective management strategies. HCPs viewed their role as including side effect management and, along with women who persisted, emphasized exercise as a key management strategy with benefits that extended beyond side effects. The importance of communication was also recognized by all groups, where breast cancer survivors focused on helpful or unhelpful ways HCPs communicated, and HCPs focused on the communication strategies they thought were helpful and identified the importance of reciprocal communication. Women who persisted with AET tended to emphasize their rationale for AET use, while women who discontinued tended to emphasize the drawbacks and unmet information needs. Similarly, HCPs viewed education as the key strategy to supporting AET use. Finally, women who discontinued AET and HCPs were both aware of patient-level contextual barriers that interfered with AET use and HCPs noted the barriers they themselves face to providing patient care.

## 4. Discussion

This qualitative descriptive study examined factors that supported and interfered with AET use among breast cancer survivors, including perspectives from women who persisted with AET, women who discontinued, and HCPs. The overlap in themes suggests widespread recognition of the most important factors affecting AET use.

When side effects, particularly arthralgia, were severe enough to impact quality of life, relationships, and day-to-day activities, they could lead participants to prioritize quality of life over quantity and discontinue AET. This is consistent with prior qualitative studies [10,11,13,14,15,24,25,26] as well as a review of qualitative studies describing a “horned dilemma” that women face in choosing between two bad options (i.e., continue AET and suffer with side effects or discontinue and fear cancer recurrence [27]). Consistent with prior literature [11,24,26,28,29], women who persisted tended to have effective strategies to manage side effects, such as lifestyle modifications, exercise, dietary changes, or supplements. The endorsement of exercise as particularly helpful for joint pain is supported by meta-analytic evidence reporting medium effect-sizes for reductions in AET-induced pain and stiffness among breast cancer survivors following exercise programs [30]. Moreover, the additional benefits of exercise (including physical health, mental health, and fatigue) identified by participants are consistent with decades of research supporting benefits of exercise for breast cancer survivors [31]. In addition to exercise, other known strategies for AET side effect management include antidepressants, acupuncture, and relaxation techniques for joint pain [32], or antidepressants, yoga, acupuncture, and environmental modification for hot flashes [33]. Providing information and improving access to strategies known to support side effect management (e.g., facilitating cancer-specific exercise programs) were suggested to support women struggling with AET use.

Consistent with reports from other studies of breast cancer survivors using AET [11,12,13,14,15], preparedness for side effects helped women cope, while unexpected side effects made women feel anxious. A lasting association between side effects and fear could develop in cases where women misinterpreted side effects as signs of metastasis or cancer recurrence. Several studies recommend thoroughly addressing AET side effects ahead of time to mitigate such associations [10,11,13,28]. Concerns may exist around the nocebo effect (i.e., adverse effects resulting from negative expectations about treatment [34]), as negative expectations about AET have been reported to predict greater side effect intensity and non-adherence over a two-year period [35]. However, evidence suggests that enhanced information, a good HCP–patient relationship, and education about coping skills can help manage negative treatment expectations [36]. Further, specifically targeting AET treatment expectations has been shown to improve expected ability to cope with side effects [37]. As several participants who discontinued AET in this study did not initially attribute their symptoms to AET, women can experience side effects uninfluenced by treatment expectations. Women should thus be prepared for potential AET side effects through proactive discussion of side effects and management strategies. Such discussions could build patients’ self-efficacy for managing side effects by informing them about behavioural strategies, pharmacological options, and the ability to switch medications. Accordingly, HCPs in this study considered informing women about side effects as important and incorporated such discussion into routine practice. Delivery of thorough, standardized information about side effects and their management by HCPs and improving access to resources such as exercise programs may be worth investigating as intervention targets. 

Another factor that distinguished women who persisted was their emphasis on reasons to justify AET use despite side effects, such as fearing cancer recurrence, not wanting to regret discontinuation in the event of a recurrence, trusting research, trusting HCPs, and being motivated by loved ones. These reasons for use are consistent with those reported by other breast cancer survivors using AET [11,14,25]. Conversely, women who discontinued tended to emphasize downsides of AET. This is unsurprising as, once a decision regarding AET persistence is made, it would be expected that participants focus on factors favoring their decision to reduce cognitive dissonance. Women who discontinued wanted more information about AET and HCPs considered education a key strategy for supporting AET use. However, provision of information should be considered necessary, but insufficient, as interventions based on education alone are generally ineffective for improving medication adherence within the context of chronic disease management [38]. This might in part explain why existing interventions that primarily involve patient education have largely failed to improve AET adherence [6,7]. It may be worth targeting attitudes toward AET without invalidating women’s experiences, for example, linking AET use to personal values, while also recognizing it can be difficult to persist with. Participants who persisted discussed how acceptance (i.e., coming to terms with one’s situation without having to like it) was helpful, which could be targeted through acceptance-based interventions. Interestingly, onset of AET-specific side effects like musculoskeletal and vasomotor symptoms has been associated with decreased cancer recurrence and increased survival [39,40,41]. This knowledge might help women attach meaning to side effects such as assurance that AET is “doing its job”. Additionally, religiosity and spirituality are associated with psychological resilience after breast cancer [42] and have been identified as factors that help women make sense of AET and persist despite side effects [43]. In exploring reasons for AET use, it would be relevant to include discussion of religion and spirituality with women who hold these beliefs.

All groups of participants highlighted the importance of communication and relationships between patients and HCPs—consistent with prior studies that have identified the importance of HCPs being caring, empathetic, responsive, and knowledgeable [11,24]; treating patients as individuals [44]; and taking their concerns seriously [13]. Farias et al. [29] interviewed 22 breast cancer survivors about physician communication and reported that communication viewed as caring, respectful, empowering decision-making, and enabling of side effect management facilitated AET persistence. In this study, HCPs similarly recognized the importance of supporting autonomy, treating women as individuals, and encouraging information-sharing through open-ended and non-judgmental questions. Training HCPs in the use of specific motivational communication strategies could be a potential target for interventions to improve AET adherence. This patient-centered, non-judgmental communication style aims to support patients in recognizing pros and cons of a behaviour and resolving ambivalence toward change [45]. A systematic review reports that training general health practitioners in motivational interviewing leads to increased use in clinical practice and improved patient health outcomes such as dietary changes [46]. 

In addition to the specific communication strategies, HCPs identified that they can only support patients insofar as patients communicate their difficulties, and that communication needs to be reciprocal to be effective. HCPs suspected that patients might be reluctant to report side effects they consider trivial or embarrassing. Similarly, some women who discontinued reported that they felt too guilty to raise concerns about AET side effects while other women were undergoing primary treatment. Issues like these could be circumvented by HCPs encouraging patient communication from the outset and reinforcing communication with responsivity and action. In addition to addressing specific content (e.g., proactive discussion of side effects, encouraging social support), Humphries et al. [28] suggested that outreach at different times along the trajectory of AET use would be helpful in responding to breast cancer survivors’ changing needs over time. Interventions to improve AET use that target providers have additional benefits of broad reach and avoid making patients solely responsible for change.

Contextual factors could make AET persistence difficult, for instance, women who worked out of financial necessity could not tolerate side effects that interfered with their ability to do their job. Although participants acknowledged that resources for breast cancer survivors that might help them cope with AET are available, women who discontinued and HCPs identified contextual factors that represented barriers to accessing such resources. Locally available exercise classes, support groups, or community programming for breast cancer survivors were only offered during daytime hours, which prevented women who worked from attending. Other predisposing barriers such as physical limitations or geographic location precluded using resources that may have helped women persist. These issues could be somewhat mitigated by increasing availability of resources through expanding programming to evenings and weekends, or including remote delivery options offering both synchronous and asynchronous content. In addition, women’s resource needs should be assessed on an ongoing basis, including during times of higher risk for discontinuation such as before discharge to family physicians.

**Figure 1:**
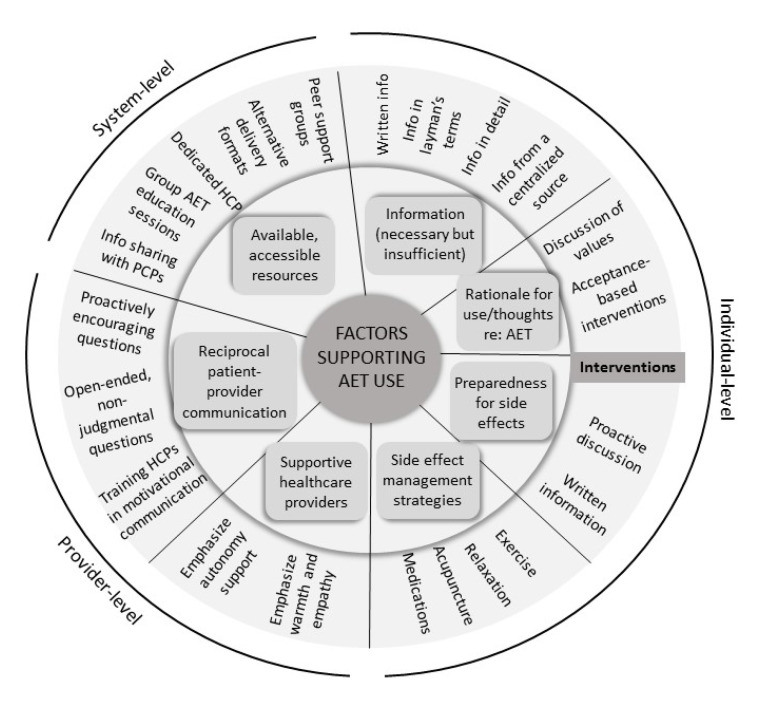
Factors supporting adjuvant endocrine therapy use and potential interventions. Factors are based on interviews with women who persisted, women who discontinued, and healthcare providers. Potential behavioural intervention components worth investigation are mapped onto each factor. Interventions could occur at the individual-, provider-, or system-level. AET = adjuvant endocrine therapy, HCP = healthcare provider, PCP = primary care provider.

It is worth investigating whether factors supporting women in AET use based on interviews with all three groups could translate into behavioural intervention components (see Figure 1). Several potential intervention components were explicitly recommended by participants. These components could be considered alone or in combination by future researchers seeking to improve AET use through behavioural intervention. Factorial studies such as those outlined in the multiphase optimization strategy could be employed to determine the most effective components or combination of components for AET use [47]. The common recognition of factors relevant to adherence across groups suggests that associated strategies for improving AET use could be relevant and acceptable to patients and providers alike.

Intervention components also exist along a continuum of intensity. In the face of limited resources, researchers could also consider triaging participants in multi-component interventions based on severity of difficulty with AET, analogous to recommendations for addressing psychosocial distress in cancer [48]. For example, healthcare organizations could at a minimum create web pages on their sites dedicated to information about AETs (i.e., mechanism, rationale, efficacy), side effects, and side effect management (e.g., evidence-based strategies for each side effect). As the quality of information about AET available online is likely to be highly variable across sources, attaching information to websites of cancer centres allows HCPs to vet the quality of information and allows patients to trust the information. As provision of information should be considered minimally necessary, but insufficient to support adherence, a moderate-intensity option might involve developing AET-specific groups for breast cancer survivors to provide mutual support and share troubleshooting tips (although participants in this study suggest that not all women would prefer this option). A more intensive AET-specific resource could be a dedicated, knowledgeable HCP within a cancer centre who could dedicate time to consulting with women individually about their AET use. Optimal decision rules to guide triaging of resources (e.g., frequency to assess difficulty with AET, criteria used to warrant change in resource intensity, best sequence of resources) could be determined through use of sequential multiple assignment randomized trial (SMART) designs [49].

### Limitations 

The first limitation of this study is the lack of ethnic diversity in this sample, which was further impacted by the exclusion of non-English speaking individuals. A more diverse sample may have provided insight into sociocultural and contextual factors that contribute to AET use that were not captured here. A second limitation is the likelihood of selection bias of HCPs, as those who volunteered to participate despite busy schedules were likely especially interested in and attuned to patient adherence. Relatedly, as breast cancer survivors were purposively sampled, there may have been implicit selection bias toward recruiting participants whose experiences aligned with the researcher’s initial beliefs about strategies to support AET use. Third, although allowing in-person and telephone interviews was meant to allow greater inclusion of participants with time and transportation constraints, it is possible that systematic differences between interview modalities (e.g., greater rapport while in-person, greater comfort with the anonymity of telephone) impacted data in ways that were undetected. Fourth, the number of participants was unbalanced across groups and the results may have differed if more women who discontinued AET and HCPs were recruited. Fifth, potentially important variables (such as health literacy or religiosity) that might have helped contextualize participant responses were not examined. Finally, it is the case that AET is not necessarily the right choice for every patient, a sentiment shared by breast cancer survivors and HCPs in this study—however, a pro-AET bias is implicit in the very topic of this project. Recommendations are thus not meant to persuade women that adherence is the right choice, but rather to support women who want to adhere, yet have difficulty doing so.

## 5. Conclusions

There was considerable overlap among the factors women who persisted, women who discontinued, and HCPs described as relevant to understanding AET use. Factors supporting women in AET use based on interviews with all three groups could be targeted by behavioural intervention components, which could be broadly acceptable (see Figure 1). Factors at the individual level include the following: education about AET (which may be necessary but insufficient alone), having a rationale for use, being prepared for side effects, and being equipped with side effect management strategies. HCP-level factors include supportive communication style and reciprocal communication with patients. Finally, available and accessible resources represents a system-level factor.

## Figures and Tables

**Table 1:** Demographic information for women who persisted (*n* = 23) and discontinued (*n* = 15).

Demographic	Women Who Persisted	Women Who Discontinued
	M(SD)	
Age	55.96 (8.48)	64.33 (7.59)
Education (years)	14.65 (2.29)	13.93 (1.64)
Years since diagnosis	2.59 (1.88)	3.67 (1.68)
	%(*n*)	
Ethnicity		
White	82.6 (19)	93.3 (14)
Asian	13.0 (3)	6.7 (1)
Mixed ethnic background	4.3 (1)	-
Cancer stage		
0	-	7.7 (1)
1	40.9 (9)	30.8 (4)
2	36.3 (8)	46.2 (6)
3	18.2 (4)	15.4 (2)
4	4.5 (1)	-
Primary treatment		
Chemotherapy	63.6 (14)	40.0 (6)
Radiotherapy	63.6 (14)	53.3 (8)
Surgery	100 (23)	66.7 (10)
Current AET		
Aromatase inhibitor	52.2 (12)	-
Tamoxifen	47.8 (11)	-
AETs used		
Aromatase inhibitor	-	26.7 (4)
Tamoxifen	-	13.3 (2)
Both	-	60.0 (9)

AET = adjuvant endocrine therapy.

**Table 2:** Additional quotes from women who persisted.

Themes (*Subthemes*)
Side Effects
***Preparedness for side effects***
“*I’m going to have [hot flashes] eventually anyway, there’s no getting away from them. Because that’s how I looked at them, I’m just going through it now and probably handling it better than I might 10 years from now*.” (Participant, age 47) “*I still keep a lot of the literature on the medication itself. Because every time a new symptom comes, it’s like, “Was that expected, should I be worried or not be worried?” And so it’s kind of nice to go back to–they did mention it and they did think that it was going to be like this*.” (Participant, age 41)
***Management strategies***
“*As I started doing these exercises I got off the internet, it was like leaps and bounds. I could finally sleep almost through the whole night. And I wasn’t in pain*.” (Participant, age 51) “*I’ve done a lot of digging, and I can’t find anything. There’s a lot of anecdotal evidence, but… I take wheatgrass, a shot of it. I do not enjoy the flavor or anything about it*.” (Participant, age 41) “*A woman I was talking to the other day, she says that every time she takes her pill, she has to remind herself of the reason that she’s choosing to do this. Whereas someone else said, "Every time I take this pill, I have to remind myself that I’m being poisoned." So there is a lot to be said for framing it*.” (Participant, age 51)
**Rationale for use**
“*I mean, obviously it helps, right? Because the cancer is hormone dependent. So, I get that it’s definitely a good thing for me to take. But I think also me understanding that helps me to take it*.” (Participant, age 51) “*I have to be strong because I’m not just making the decision for myself, I’m making it for my family. In my mind it’s like, “Do I want to commit suicide or do I want to live?” I can’t be selfish because I’ve got a sore arm or a sore knee*.” (Participant, age 51) “*I think it’s saving my life [laughs], saving the cancer from coming back. I feel like I have that mindset so I wouldn’t think twice, no matter what the side effects were*.” (Participant, age 70)
**Experiences with the Healthcare System**
***Interactions with healthcare providers***
“*[The oncologist] just said, "You know what? You just have to give it a try, because it’s your protector. I know it’s brutal," but he said, "Just think of it as your protector." So it was totally how he delivered, absolutely*.” (Participant, age 59) “*[The oncologist] certainly left it in my ballpark, he never forced me to but he definitely talked to me about the pros and the cons. Yeah, he handled it really well*”. (Participant, age 62) “*I did not contact my oncologist about this, because I don’t feel like they listen to me anyway, so I just made my own decision*.” (Participant, age 52)
***Availability of resources***
“*There’s so much out there. There’s support groups, there’s people to talk to, there’s information, we have the internet. It’s there if you want it, I don’t think there needs to be more*.” (Participant, age 47) “*I’m pretty open to the idea of counseling–I think counseling was just something that was offered right upon diagnosis*.” (Participant, age 51)

**Table 3:** Additional quotes from women who discontinued.

Themes (*Subthemes*)
Unmanageable side effects
“*Because there was so much pain associated, I didn’t want to be touched by anybody, so then it affects all your relationships, right*?” (Participant, age 59) “*I decided I wanted to be able to golf and ski and do whatever without aching and feeling miserable. As I said, a shorter, better life than a longer one that wasn’t so good*.” (Participant, age 77) “*I tried many types and I have prescriptions for everything that I could possibly get to try to help that body pain and nothing works. With the result that I ended up with upper gut issues that I’m only just starting to be able to investigate now*.” (Participant, age 50)
**Reluctance**
***Information needs***
“*I think having more support on the drug side. Having more percentages and more data on Tamoxifen. How many people it’s worked for, how many people it hasn’t worked for, success rates, things like that. It just seemed like it was expected, and it didn’t seem that there was a lot of information that they could give me on the benefits of Tamoxifen. It was just kind of like “Here you go*”. (Participant, age 68) “*I think people assume that you understand what they’re talking about, but you really don’t, in layman’s terms. So, I can say that was a bit of a lack of information at that point*.” (Participant, age 64)
***Drawbacks of adjuvant endocrine therapy***
“*There is also all the research on women that had been taking it for umpteen years and still getting [cancer] recurrences. So, it just didn’t make any sense that I would have to feel that rotten if they couldn’t even guarantee that it would actually work*.” (Participant, age 50) “*To me Tamoxifen falls into the category of a pill for every ill. If you’ve got estrogen receptor positive breast cancer, they prescribe Tamoxifen for you, not even taking into any other factors into consideration*.” (Participant, age 68)
**Experiences with Healthcare Providers**
“*They did their best to make sure that I had everything that they could think of. It wasn’t their fault, and it wasn’t that they didn’t try hard. They sure did*.” (Participant, age 67) “*I would have a whole bunch of questions written down when I would go in to see [oncologist]. I’d say, “Okay, I have a list of questions for you”. He would say things like “Well, how many?”, and stuff like that. So, I just found him quite abrupt, and not somebody that I really want on my healthcare team*.” (Participant, age 68) “*I knew that [AET use] was always up to me, and I was thankful for the information that they provided. But also thankful for the fact that they left the decision up to me*.” (Participant, age 67)
**Contextual Factors**
“*Probably at 40, I would’ve had kids and at 69, I don’t have a lot of responsibilities. You know, I’m over the hill and so I didn’t feel the need to struggle with it as the same as I would’ve if I was younger*.” (Participant, age 69) “*So out of financial concern, I was working fulltime. The financial concern was causing me to stop taking my medication early, just in order to keep my job*.” (Participant, age 57) “*Having [cancer] come back a second time-I was more open to taking medications if they were going to be helpful*.” (Participant, age 64)

**Table 4:** Additional quotes from healthcare providers.

Themes
Barriers at Patient-and System-Level
“*In theory, cost shouldn’t be a barrier because the medications are provided free of charge, but logistically there is lots of costs. Patients have to travel, like driving in from a ranch. It’s a lot of money and gas*.” (Pharmacist) “*Unless they’re calling for a refill, there would be no way of following them closely, so we do have a certain percentage of patients that are lost through this*.” (Pharmacist) “*The other thing that I think is helpful, and it can be harmful for people, is the internet too. There are a lot of chat groups, these sort of things, where good information but, sometimes wrong information, misinformation is being communicated to individuals. As things get a little bit more electronic based, I spend a lot more time correcting misinformation than I did ever before*.” (Oncologist)
**Side Effect Management**
“*Light to moderate or weight bearing exercise is really good. So non-pharmacological measures first. If that doesn’t work, then of course pharmacological measures, such as using Tylenol Arthritis or anti-inflammatories if necessary*.” (Pharmacist) “*I think the fact that women after breast cancer have a lot of issues not only with the side effects of medication but also the feeling of isolation, depression sometimes, grieving, the fact that they often struggle with weight. They may have struggled with weight as a risk factor for their cancer. I think that the idea of having a more coordinated approach to offer exercise and coaching essentially for physical health would be another significant help*.” (Oncologist) “*But if [side effects] get actually worrisome, we usually use venlafaxine and sometimes SSRIs. That’s only for when they have pretty much bothersome hot flashes*.” (Oncology resident)
**Patient-Provider Interactions**
“*So I try and give people things that they can actually do. Because I feel like that’s a little more empowering, rather than just saying “Hey, here’s the medication*.” (Oncology resident) “*I feel that part of it will also come down to how you present it initially. So part of my initial talk to [patients] is like, “I’m gonna see you in 3 months after I start it, but I really want you to call me if you notice anything.” And I tend to try and get my patients to err on the side of calling rather than not calling*.” (Oncology resident) “*There’s two type of patients. There is one who won’t say anything and struggle on and struggle on and doesn’t let the clinic know. There is another patient who will very quickly verbalize that and need help or demand a change, because you can’t tolerate it, it’s affected her quality of life*.” (Pharmacist) “*Although there’s no food restrictions, with food on any of these agents, I’m a big proponent of–we have to keep it simple. The more restrictions and complications that we add, we risk people not taking it, and then of course we’ve lost all potential benefits of taking it*.” (Pharmacist)

**Table 5:** Comparison of themes across groups.

Themes	Women Who Persisted with AET (*n* = 23)	Women Who Discontinued AET (*n* = 15)	Healthcare Providers Who Manage AET (*n* = 9)
Side effects	Women were prepared to deal with side effects	Side effects were overwhelming and interfered with quality of life	Side effects are a key factor associated with AET discontinuation
Side effect management strategies	Women had strategies to successfully manage side effects (e.g., exercise)	Women were unable to find effective side effect management strategies	HCPs recommended behavioural (e.g., exercise) and pharmacological strategies
Information		Women wanted more, detailed, relevant, and understandable information	HCPs viewed providing information as a key strategy for promoting AET use
Thoughts related to AET use	Women emphasized the life-saving potential of AET and personal reasons for use	Women emphasized that AET was not guaranteed to work and feared long-term effects (e.g., bone loss)	HCPs recognized patients could have pre-existing negative bias toward AET
Communication with healthcare providers	Supportive HCPs could be a key factor in continuing AET	Women appreciated autonomy; they were frustrated when they perceived HCPs as rushed or un-responsive	Communication needs to be reciprocal to be effective
Experiences with the healthcare system	Resources were available to support women in AET use		Healthcare system constraints make it difficult for HCPs to address concerns thoroughly
Contextual factors		Being older, pre-existing illness, and having to work could make AET seem less worthwhile	Peripheral out-of-pocket costs could still impact AET use Offering mailed prescriptions programs could help

AET = adjuvant endocrine therapy, HCP = healthcare provider.

## Data Availability

Data are not publicly available to protect confidentiality of participant information.

## Appendix A. Qualitative Interview Guide

Guide for breast cancer survivors (either persistent or non-persistent):

1. Tell me a bit about your cancer story—when did you find out?
  - Probe: How was treatment for you?
  - Probe: How have you been since?
2. (Knowledge): I understand your doctor has prescribed this [medication] for you. Can you tell me why they prescribed this medication? What is its purpose?
  - Probe: Where did you get your information about [name of drug] from?
  - Probe: How did you feel about the information you got?
3. (General experience with use): What has taking [medication] been like for you?
  - Probe: What stands out about the experience the most?
  - Probe: How was it compared to how you expected it would be?
  - Probe: Is there anything you like about taking [name of drug]? Anything you dislike?
4. (Decisions about use): Sometimes people decide not to take oral medications like these for breast cancer treatment. What are your plans for taking/why do you keep taking/why did you stop taking [medication]?
  - Probe: What helped you to decide to keep taking it?
  - Probe: What made you decide to stop taking it?
5. (Barriers): What are some things that you think might make it difficult/has anything made it difficult to take [medication]?
  - Probe: What are some things that might make it hard for other women to take [name of drug]?
6. (Facilitators): What are some things you think might help you take/keep taking/restart [medication]?
  - Probe: Was there anything that would have been helpful?
  - Probe: What are some things that might make it easier for other women to take [name of drug]?
7. (Intervention preferences):
  - For all: One thing we are interested in doing is helping women who want to take their oral cancer therapies but are having trouble doing so. Based on your experience what kind of professional support or any support in general do you think would be helpful?
  - For all: What would be your preferred way to communicate with someone who wanted to help you take your medications (e.g., phone, e-mail, in person)? Why?

Guide for healthcare providers:

1. To get things started, I was wondering if you could tell me a little bit about your practice… how do you spend your time?
2. (Knowledge): What sort of information do you provide your breast cancer patients when you prescribe adjuvant endocrine therapies for them?
  - Follow-up: What other places do your patients get information from?
  - Follow-up: What is your sense of the accuracy of the knowledge most patients have about these medications?
3. Question 3 (general experience with use): How do your patients typically feel about taking adjuvant endocrine therapies?
  - Follow-up: What do they like? What do they dislike?
4. Question 4 (use): In your experience, how many women take them as prescribed?
5. Question 5 (barriers): What are some things that make it difficult for women to take these medications?
6. Question 5 (facilitators): What are some things that make it easier for women to take these medications?
7. Question 6 (interventions): Have you ever tried strategies to help women adhere to prescribed adjuvant endocrine therapies?
  - Follow-up: What have you tried?
  - Follow-up: What has worked in the past, what hasn’t worked?
